# Dual-Mode Triboelectric and Capacitive Pressure Sensor Based on Anodic Aluminum Oxide

**DOI:** 10.3390/nano16120771

**Published:** 2026-06-19

**Authors:** Chung-Yu Yu, Chia-Wei Hung, Chin-An Ku, Geng-Fu Li, Cheng-Hao Chiu, Chen-Kuei Chung

**Affiliations:** Department of Mechanical Engineering, National Cheng Kung University, Tainan 701, Taiwan

**Keywords:** dual-mode sensor, TENG, AAO, pressure sensor, capacitive sensor

## Abstract

Triboelectric nanogenerators (TENG) show significant potential in pressure sensing by converting mechanical disturbances into electrical signals positively correlated with the magnitude of the applied force, yet their development as practical pressure sensors is severely hindered by the major drawback of only detecting transient mechanical inputs. Additionally, traditional dual-mode pressure sensors have typically required complex multilayer structures and time-consuming fabrication processes. Here, a simple dual-mode pressure sensor of novel structure integrated with TENG and anodic aluminum oxide (AAO) for both dynamic and static pressure detection is proposed. Nanoporous AAO is directly grown on an aluminum substrate to simplify the traditionally complex multi-layer structure of dual-mode pressure sensors. The AAO layer serves a dual functionality by acting as an active triboelectric layer that significantly enhances the triboelectric output performance while concurrently functioning as the capacitive dielectric layer. A polydimethylsiloxane (PDMS) film is employed as the elastic counterpart to pair with the AAO substrate. The influence of PDMS thickness on the charge accumulation and extraction of the TENG mode is investigated to optimize the device output. Under optimal configurations, the streamlined Al-AAO/PDMS sensor demonstrates good sensitivity and linearity (R^2^ > 0.99) for both dynamic triboelectric voltage (1.05 V/kPa) and static capacitance (5.56 pF/kPa) over a wide sensing range of 1–73 kPa. This dual-mode sensor effectively overcomes the transient limitation of conventional single-mode TENGs and shows significant potential for future smart tactile applications.

## 1. Introduction

With the rapid rise of the Internet of Things (IoT) and wearable electronic devices, the development of pressure sensors that are capable of converting ambient mechanical energy into electricity has become a significant research focus [1,2,3,4]. Among various technologies, the triboelectric nanogenerator (TENG) has demonstrated immense potential due to its high output voltage, potential self-powered characteristics, and excellent dynamic response capabilities [5,6,7]. The performance of a TENG originates from the coupling effect of contact electrification and electrostatic induction, as a single-mode dynamic pressure sensor [8,9]. Therefore, TENG electrical signals are generated only during the dynamic processes of contact or separation. Once the external force enters a static state under constant pressure, the charge transfer ceases, resulting in signal interruption [10,11]. This inherent characteristic severely restricts the application of TENGs in environments requiring sustained static pressure monitoring, presenting a critical bottleneck for tactile sensing [12]. To overcome this limitation, hybrid mechanical sensors that integrate TENG with capacitive sensing have emerged as a prominent research trend in recent years [13,14,15]. These systems utilize TENG for active transient detection while incorporating capacitive technology to achieve precise static mechanical measurement. However, a critical research gap still persists in existing dual-mode TENG-capacitive sensors, which is primarily manifested in two aspects. First, from an architectural standpoint, current dual-mode devices rely on highly complex, multi-layered structures consisting of four or more functional layers, which inevitably increases device thickness and the risk of interfacial delamination. Second, despite their complex designs, many conventional dual-mode configurations still exhibit suboptimal sensing sensitivity, failing to provide high-fidelity readings under both dynamic and static loading states due to poor material coupling [13,14,15]. For instance, Li et al. utilized PVDF and graphene as the triboelectric and electrode layers, respectively, while incorporating an additional polydimethylsiloxane (PDMS)/graphene combination to form the capacitive component [13]. The resulting four-layer composite structure achieved a sensitivity of 0.4485 kPa^−1^ [13]. Similarly, Fang et al. reported a five-layer configuration consisting of two polyethylene terephthalate (PET) films sandwiching two transparent indium tin oxide (ITO) layers and one PDMS layer to form two single-electrode TENGs and a capacitor [14]. This device exhibited sensitivities of 0.25 kPa^−1^ in the high-pressure regime [14]. Such structural complexity entails intricate and tedious fabrication processes, posing significant challenges for practical implementation and scalability. Consequently, developing a streamlined dual-mode pressure sensor that simplifies the structural layout, shortens fabrication time, and delivers superior sensitivity remains a significant challenge. In addition to processing efficiency, a distinct research gap exists regarding the capability of conventional single-mode TENG material systems to achieve dual-mode functionality. Among conventional TENGs characterized by simple fabrication processes and designed strictly for dynamic sensing, the utilization of PDMS paired with aluminum represents a widely adopted choice [16,17,18,19,20]. Nevertheless, a notable limitation resides in the fact that these conventional, pure Al/PDMS TENG systems inherently lack a viable structural framework or design suitable for static capacitive sensing. Consequently, the potential of expanding these standard TENG material pairs to concurrently manage both dynamic contact electrification and static dielectric modulation without stacking several functional layers remains unaddressed. To introduce a viable structural framework for capacitive sensing into the aluminum-based system, the integration of nanoporous anodic aluminum oxide (AAO) represents a highly effective strategy [21,22]. AAO is a self-organized nanostructure characterized by a high-density array of vertical nanopores [22]. Conventionally, the AAO layer functions as a static capacitive element by establishing a parallel-plate capacitor configuration [22,23]. While aluminum-based configurations have been extensively investigated for dynamic sensing in TENG, and nanoporous AAO structures have been explored for static capacitive modulations, the synergistic integration of these two distinct mechanisms into a unified Al-AAO hybrid framework for dual-mode triboelectric and capacitive pressure sensing remains completely unaddressed.

Here, a dual-mode pressure sensor integrated with TENG and AAO for dynamic and static pressure detection is proposed. In contrast to traditional complex designs comprising four or more layers, this work utilizes an Al-AAO/PDMS bilayer configuration to achieve a dual-mode triboelectric and capacitive sensing architecture, significantly reducing fabrication time while streamlining the sensor system’s construction. Nanoporous AAO is directly grown on an aluminum substrate to serve as both the triboelectric layer and the capacitive sensing element. Furthermore, the aluminum substrate employed for AAO growth functions directly as the conductive electrode. Compared with the traditional Al/PDMS triboelectric pair, the proposed structure retains the same inherent simplicity, with the primary distinction being the nanoporous AAO architecture integrated onto the aluminum base. The AAO serves as a triboelectric layer because its intrinsic nanoporous morphology provides a highly texturized topography, which substantially enhances the effective contact area and promotes contact electrification when paired with a soft counterpart. The porous characteristics of AAO allow PDMS to deform into the pores under pressure, further contributing to the capacitance change. By integrating TENG and AAO into a hybrid capacitive sensor, this design can overcome the limitation of TENGs in measuring both dynamic and static pressure. Owing to its dual-mode sensing capability, structural simplicity, and rapid one-step anodization process, this Al-AAO/PDMS dual-mode pressure sensor demonstrates significant potential for pulse wave detection [24], tactile discrimination [25], robotic hands [26], foot pressure distribution monitoring [27], and impact force detection during locomotion [28].

## 2. Materials and Methods

### 2.1. Materials

The 1050 aluminum alloy (AA1050) was obtained from Fapo Enterprise, Taipei, Taiwan. The AA1050 is composed of 99.5% aluminum, 0.3% iron, and 0.03% copper, with trace impurities including magnesium, manganese, silicon, titanium, vanadium, and zinc. The base and curing agent of the PDMS (Sylgard 184, Dow Corning, Midland, MI, USA) was purchased from Sigma-Aldrich (St. Louis, MO, USA). Commercial insulating tape and double-sided conductive copper foil tape (10 mm in width) were used as received. The platinum target (99.99% purity, 57 mm in diameter, and 0.1 mm in thickness) was purchased from Jie Dong Co., Ltd., Tainan City, Taiwan. The oxalic acid (≥99.5% purity) was purchased from Miami Chemical Co., Ltd., Tainan City, Taiwan. Deionized (DI) water was used to prepare oxalic acid aqueous solutions throughout the experiments.

### 2.2. Fabrication of Al-AAO/PDMS Device

**Figure 1:**
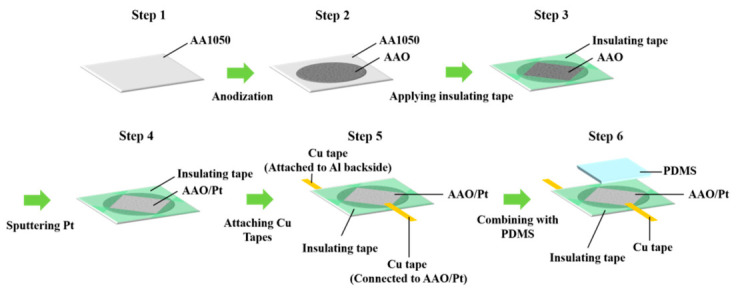
The process flow for dual-mode Al-AAO/PDMS pressure sensor fabrication.

The process flow of Al-AAO/PDMS pressure sensor preparation is shown in Figure 1. In step 1, a commercial AA1050 substrate with a thickness of 1 mm was cut into an appropriate size (2.5 cm × 2.5 cm). In step 2, anodization was performed in a 0.3 M aqueous oxalic acid solution at 25 °C with the hybrid pulse anodization (HPA) [22] technique at 40/−2 V with a duty ratio of 5 s/5 s for 1 h. For the anodization process, a potentiostat (EC5000, Jiehan, Taichung City, Taiwan) with a three-electrode system was used with platinum mesh as the counter electrode, 1050 aluminum alloy in our self-made holder as the working electrode, and calomel as the reference electrode. The self-made holder restricted the exposed and anodized region of the aluminum substrate to a circular area with a diameter of 2 cm. In step 3, insulating tape was applied to mask the peripheral regions, defining a central exposed square active area with a side length of 1.4 cm. This area was actually used for the subsequent measurements. This was to prevent the surrounding aluminum substrate from coming into direct contact with the PDMS. In step 4, a platinum layer was then sputtered onto the AAO membrane with a thickness of 10 nm using a sputter coater (JEC-3000FC, JEOL, Tokyo, Japan). Because this platinum layer is extremely thin, a highly conformal conductive coating is formed along the top surface and the inner pore walls of the AAO membrane, rather than creating a thick, planarizing blockage over the openings. Consequently, the original nanoporous topography and pore accessibility of the underlying AAO are successfully preserved. In step 5, copper tapes were attached to the device to serve as conductive wires for subsequent electrical measurements. Specifically, one Cu tape was attached to the Al backside of the substrate, and the other Cu tape was connected directly to the sputtered AAO/Pt layer. Separately, the flat PDMS was prepared by casting using a polymethyl methacrylate (PMMA) mold, with its thickness controlled at 150, 300, 450, and 600 μm for comparison. The PDMS was fabricated by mixing base and curing agent at a ratio of 10:1 and put in a vacuum chamber with a pressure of 500 mTorr for 10 min, followed by curing at 75 °C for 1 h. Finally, in step 6, after demolding, the PDMS film was placed onto the Pt-sputtered AAO substrate, completing the sensor fabrication. The actual optical photograph of the fully assembled dual-mode Al-AAO/PDMS pressure sensor was shown in Figure S1. The aluminum substrate and the top platinum layer on the AAO serve as two parallel metal electrodes for the capacitor, with the copper tape serving solely as a conductive wire to facilitate subsequent electrical measurements. Meanwhile, the AAO and PDMS act as the two triboelectric layers for the TENG. In addition, the AAO also functions as a porous dielectric layer for the capacitor, allowing the PDMS to penetrate the pores and modulate the capacitance.

### 2.3. Characterization and Measurement

The morphology of AAO was observed with a high-resolution field-emission scanning electron microscope (HRFESEM, HITACHI, SU-5000, Tokyo, Japan), and then the nanopores were analyzed by ImageJ software (version 1.43u). The open-circuit voltage and short-circuit current of the fabricated TENG device were characterized under controlled mechanical stimulation profiles. Periodic vertical impact forces were delivered to the center of the active device area using a pneumatic cylinder (D:S-PAZ-DW20-100PPV cylinder, FESTO, Esslingen am Neckar, Germany). To ensure rigorous testing conditions, the cyclic loading was performed with a fixed peak force of 15 N operating at a dynamic frequency of 7 Hz, which corresponds to an applied peak pressure of approximately 76.53 kPa given the 1.96 cm^2^ active area. Furthermore, all electrical measurements were conducted in a standard air-conditioned laboratory at an ambient temperature of approximately 25 °C and a relative humidity of around 60% to maintain steady environmental conditions. The contact/separation distance of the Al-AAO/PDMS TENG was 15 mm. The LED test was also conducted under the same condition. The open-circuit voltage, short-circuit current, and power under different loads were measured by a Keithley DMM6500 (Solon, OH, USA). The capacitance variations in the Al-AAO/PDMS device under an external mechanical load, provided by a standard weight, were measured by an LCR meter (HIOKI 3522-50, Nagano, Japan) applying a stable AC probe voltage of ±1 V. The excitation frequency for the capacitance characterization was fixed at 1000 Hz. In this specific laboratory stage, to evaluate the fundamental performance of each mode without cross-talk artifacts, the dynamic TENG curves and static capacitance curves were measured using independent platforms (Keithley DMM6500 and LCR meter). This was implemented to ensure that the characterization of one mode was not affected by the electrical noise from the other. To ensure statistical validity and eliminate transient signal artifacts, each reported dynamic electrical value (open-circuit voltage and short-circuit current) was obtained by averaging 21 consecutive measurement peaks under cyclic loading. Additionally, the long-term operational reproducibility and mechanical stability of the sensor architecture were evaluated via a continuous durability test of 5000 cycles.

## 3. Results

**Figure 2:**
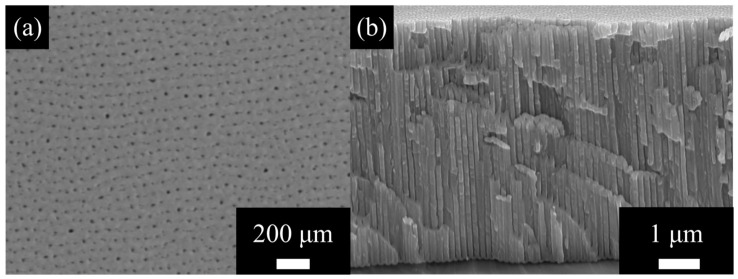
The SEM images of (**a**) top-view and (**b**) cross-section view of AAO prepared by HPA at 40/−2 V at 25 °C for 1 h.

Figure 2a and Figure 2b respectively show the top-view and cross-sectional SEM images of AA1050 anodized using the HPA method at 40/−2 V for 1 h at 25 °C. The average pore diameter is 31.45 nm, and the film thickness is 5.9 µm. To ensure dual-mode sensing performance, maintaining a well-preserved and complete nanoporous structure across the active layer is essential. As shown in Figure 2, although the anodization process was carried out at a temperature of 25 °C, the detrimental phenomenon of pore burning did not occur. This successful preservation of pore integrity is primarily attributed to the HPA technique. By applying alternating negative potentials during the square-wave voltage cycles, the electrical current periodically drops to zero, which effectively mitigates localized Joule heat accumulation. As a result, the anodization temperature can be increased to 25 °C while still maintaining pore integrity without burning [22]. Consequently, the resulting unburned, highly textured nanoporous matrix successfully maintains its structural integrity at elevated temperatures. This optimized framework is critical to maximizing the effective contact area for dynamic contact electrification while simultaneously providing accessible nano-channels for static polymer-penetration-driven capacitance modulations.

**Figure 3:**
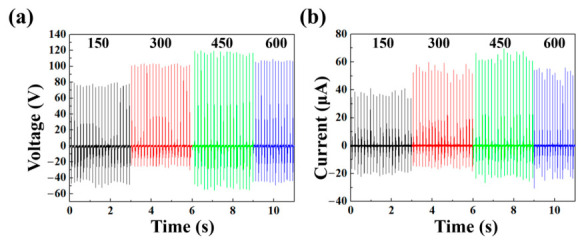
(**a**) Open-circuit voltage and (**b**) short-circuit current of the Al-AAO/PDMS devices with PDMS thicknesses of 150, 300, 450, and 600 µm.

Figure 3a,b show the open-circuit voltage and short-circuit current of Al-AAO/PDMS TENGs with different PDMS thicknesses of 150 to 600 µm. As the PDMS thickness increases from 150 to 450 µm, both the voltage and current exhibit an increasing trend, reaching maximum values of 118.4 V and 66.2 µA at 450 µm. This enhancement can be attributed to charge accumulation. PDMS, as a dielectric layer, has strong charge-storage capability, and a thicker PDMS layer allows more charges to accumulate, thereby increasing the open-circuit voltage and short-circuit current. However, when the thickness exceeds a certain threshold, the resistance for the accumulated charges to be extracted by the aluminum electrode becomes larger, reducing the power-generation performance. As a result, both the open-circuit voltage and short-circuit current decrease for the 600 µm PDMS sample. The mechanism governing the influence of PDMS thickness on TENG performance relies on the competition between charge accumulation and charge extraction, which leads to an optimal thickness that maximizes the electrical output. In this study, the TENG configuration with a PDMS thickness of 450 µm exhibited the best performance, and therefore, this specific thickness was selected for subsequent experiments.

**Figure 4:**
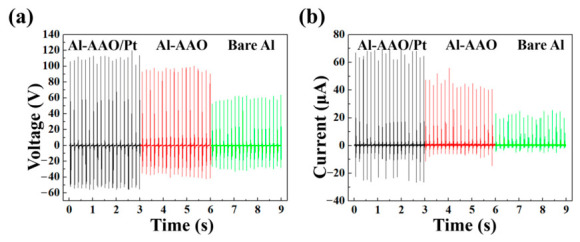
Comparison of (**a**) open-circuit voltage and (**b**) short-circuit current for TENG devices utilizing Al-AAO/Pt, Al-AAO, and bare Al layers paired with PDMS.

To systematically elucidate the enhancement mechanism of the nanoporous AAO structure and the sputtered Pt thin film on the triboelectric output performance, a comprehensive control experiment was conducted. With the optimized configuration (450-μm PDMS), the electrical outputs of the Al-AAO/Pt/PDMS sensor were systematically compared with those of the Al-AAO/PDMS (without Pt) and conventional bare Al/PDMS sensors under identical testing conditions. Figure 4 shows a comparison of (a) open-circuit voltage and (b) short-circuit current for TENG devices utilizing Al-AAO/Pt, Al-AAO, and bare Al layers paired with PDMS. As illustrated in Figure 4a,b, the conventional bare Al/PDMS configuration delivered the lowest open-circuit voltage and short-circuit current of 58.4 V and 21.2 μA, respectively. In contrast, the integration of the nanoporous AAO layer (Al-AAO/PDMS) led to a significant increase in both open-circuit voltage (to 98.3 V) and short-circuit current (to 49.7 μA). This substantial enhancement is primarily attributed to the intrinsic nanoporous morphology of the AAO, which delivers a highly texturized topography that drastically expands the effective contact area and facilitates contact electrification when paired with the soft PDMS counterpart. Furthermore, upon the deposition of the 10 nm Pt layer (Al-AAO/Pt/PDMS), the electrical output performance exhibited an additional slight enhancement. Finally, the open-circuit voltage and short-circuit current achieved 118.4 V and 66.2 μA, respectively. This improvement is due to the ultra-thin Pt layer acting as a highly conductive interface that optimizes charge induction and extraction efficiency across the active boundary. Crucially, beyond this output boosting, the introduction of the conformal Pt-coated AAO framework provides a viable structural architecture for parallel-plate capacitive sensing, thereby successfully enabling the single-device dual-mode capability without the necessity of piling extra functional layers. This control matrix firmly substantiates the structural rationale and synergistic advantages of our proposed bilayer design. This proposed architecture cleverly utilizes the AAO as both the triboelectric layer of the TENG and the dielectric layer of the capacitor, representing the core innovation of this study in simplifying traditional multilayer structures.

**Figure 5:**
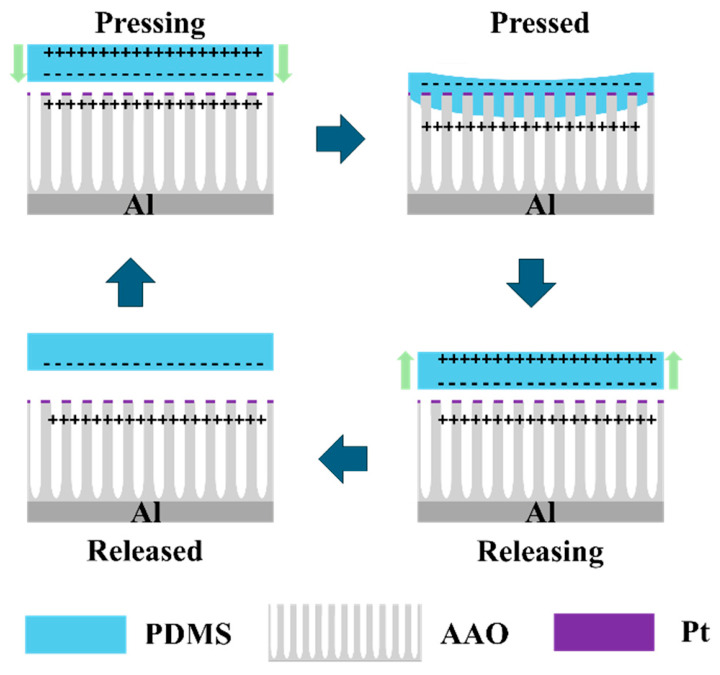
Schematic diagram of the cyclic operation of the vertical contact-separation Al-AAO/PDMS device and its charge distribution. The blue arrows indicate the cyclic process of vertical contact-separation, while the green arrows show the moving direction of the PDMS film. The -/+ symbols represent the negative and positive charges, respectively.

Figure 5 shows the schematic diagram of the cyclic operation of the vertical contact-separation Al-AAO/PDMS device and its charge distribution. The continuous mechanical working cycle is divided into four distinct sequential stages to elucidate both the dynamic triboelectric and static capacitive sensing mechanisms. In the initial “pressing” state, an applied external load drives the negatively charged PDMS film downward toward the platinum-coated AAO substrate. As the bottom surface of the polymer makes contact with the top of the AAO template, contact electrification is initiated, which induces positive charges on the surface of the AAO owing to the intimate proximity to the negative triboelectric surface charges of the deforming PDMS layer. When the external load reaches its peak, the device enters the fully “pressed” state, where the elastic PDMS film undergoes conformal deformation and deeply penetrates the restricted cylindrical AAO nanopores. This physical penetration maximizes the effective contact area between the triboelectric pair, thereby securing the maximum density of separated surface charges for the triboelectric nanogenerator mode. Concurrently, this mechanical deformation drives the polymer to displace the internal air within the nanoporous channels. Because the dielectric constant of the entering PDMS is significantly higher than that of the displaced air, this maximum penetration behavior alters the composite dielectric properties and induces the peak capacitance output, which successfully validates the static sensing capability of the device. As the mechanical pressure is subsequently withdrawn, the sensor enters the “releasing” state, causing the elastic PDMS film to begin separating from the platinum-coated AAO template. This physical separation disrupts the electrostatic equilibrium at the contact interface, which generates a reverse potential gradient and drives an electron flow through the external circuit to produce a dynamic electrical output signal. Finally, upon the complete removal of the external force, the device reaches the fully “released” state. In this condition, the PDMS film returns to its original undeformed profile entirely apart from the substrate. The current flow ceases completely as the electrostatic charges stabilize, and the capacitance drops back to its baseline value due to the complete withdrawal of the polymer from the nano-channels. This four-stage sequential process can be continuously repeated to deliver stable dynamic voltage pulses alongside reliable static capacitance modulations within a single bilayer configuration.

**Figure 6:**
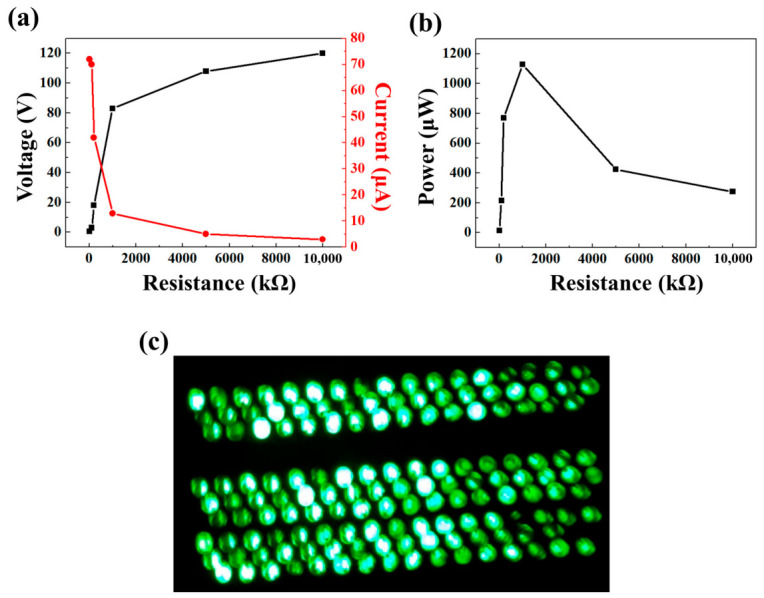
(**a**) Voltage and current values of the Al-AAO/PDMS device with a PDMS thickness of 450 μm under different load resistances, (**b**) maximum output power of 1129.2 μW at 1000 kΩ, and (**c**) 150 LEDs illuminated by the TENG with an Al-AAO/PDMS structure.

Figure 6a shows the output voltage and current of the Al-AAO/PDMS device under different external loads, while Figure 6b presents the corresponding power calculated from the output voltage and current. The resistances used were 1, 10, 100, 1000, 5000, and 10,000 kΩ. As the external load resistance increases from 1 kΩ to 10,000 kΩ, the output voltage exhibits a continuous upward trend while the current drops drastically. This behavior is governed by Ohm’s law and the internal polarization characteristics of the device where low external resistance values approach short-circuit conditions that maximize electron transfer and minimize voltage drop across the load. Conversely, elevated load resistances restrict the flow of charges through the external circuit, which leads to a massive accumulation of potential difference across the resistor at the cost of current amplitude. Consequently, the instantaneous peak power reaches its maximum value of 1129.2 µW at an optimal load resistance of 1000 kΩ. This distinctive peak signifies the state of ideal impedance matching where the external load resistance perfectly balances the inherent internal impedance of the Al-AAO/PDMS triboelectric nanogenerator system, thereby yielding a maximum power density of 5.76 W/m^2^. To further demonstrate the practical significance of this optimized power output, the generated electrical signals were utilized to directly power 150 commercial green LEDs (Nan-I Electronics Co., Ltd., Tainan, Taiwan), as depicted in Figure 6c. The successful illumination of a 150-LED array proves the energy-harvesting efficiency and real-world feasibility of our dual-mode tactile sensor for self-powered microelectronics.

**Figure 7:**
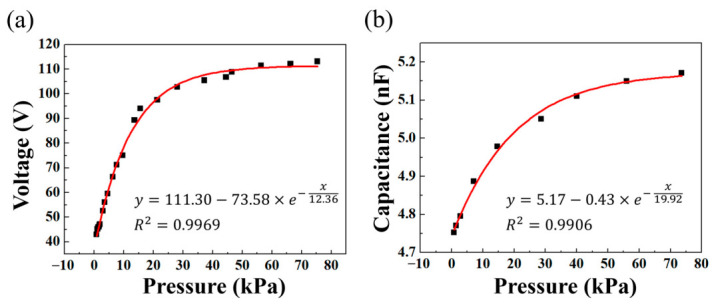
Pressure-sensing capabilities of the dual-mode Al-AAO/PDMS device under (**a**) dynamic contact-and-separation testing and (**b**) static pressure loading. The black squares denote the data points, and the red lines indicate the fitting curves.

Figure 7a and Figure 7b show the dynamic and static pressure sensing capabilities, respectively, of the Al-AAO/PDMS pressure sensor, with a measurement range of 1 to 73 kPa. Figure 7a presents the TENG voltage signals obtained by rapidly tapping the device under different pressures from 1 to 73 kPa with a fixed operating frequency of 7 Hz and a contact/separation distance of 15 mm. The output increases from 42.9 V at 1 kPa to 118.4 V at 73 kPa. By exponential fitting, the measured values show excellent agreement with the fitted curve, with a correlation coefficient (R^2^) of 0.9969. This indicates that effective sensing can be achieved across the pressure range of 1 to 73 kPa. On the other hand, Figure 7b shows the capacitance values of the Al-AAO/PDMS sensor under different pressures by placing standard weights. The signal increases from 4.77 nF at 1 kPa to 5.17 nF at 73 kPa. By data fitting with an exponential function, the measured capacitance values agree closely with the fitted curve, with an R^2^ of 0.9906. This also indicates that effective sensing can be achieved within the pressure range of 1 to 73 kPa.

Although the direct in situ cross-sectional visualization of the PDMS penetration into the AAO nanopores under dynamic pressure is restricted by the immediate elastic recovery of the polymer during sample preparation, this microscopic mechanism is strongly validated by the macroscopic voltage and capacitive response. In Figure 7a,b, a critical feature of these two characteristic curves is the synchronized saturation behavior observed in both the triboelectric voltage and the static capacitance at 73 kPa. This synchronization provides definitive empirical proof of the single-interface dual-mode sensing mechanism because both electrical signals are governed by the exact same micro-mechanical interface deformation. As the external pressure rises, the gradual penetration of the elastic polymer into the rigid nanopores simultaneously expands the effective contact area for dynamic electrification and displaces the internal air for static dielectric modulation. Once the polymer penetration reaches its geometric and elastic limit within the restricted nano-channels, further deformation is constrained, which causes both the voltage output and the capacitance change to flatten into a matching plateau state at 73 kPa.

Furthermore, because both the voltage and capacitance responses follow an exponential growth function, the instantaneous sensitivity of the hybrid device varies across different operating regimes. In the low-pressure regime below 20 kPa, both curves exhibit a steep slope, which yields an exceptionally high sensitivity that is optimal for detecting subtle tactile stimuli and weak physiological signals. In the higher-pressure regime approaching 73 kPa, the mechanical resistance arising from the nano-confined air compression and the rigidity of the oxide pore walls leads to a progressive reduction in the response slope, which naturally transitions the device into the stable saturation zone.

The sensitivity of TENG sensing is defined as the change in signal per unit of pressure [24], as shown in Equation (1) below:

(1) $$Sensitivity=\;\frac{V-V_{0}}{P-P_{0}}$$

where *V* and *P* represent the voltage and pressure at a given measurement point, and *V*_0_ and *P*_0_ represent the voltage and pressure at the minimum measured pressure. Based on Equation (1), the proposed Al-AAO/PDMS pressure sensor exhibits a good sensitivity of 1.05 V/kPa up to 73 kPa.

On the other hand, the Al-AAO/Pt structure can be described as a parallel-plate capacitor [21], as shown in Equation (2):

(2) $$C=\;\frac{\varepsilon A}{d}$$

where ε is the dielectric constant of AAO, A is the electrode area, which is defined by the Pt-sputtered area in this case, and d is the dielectric thickness, or the thickness of the AAO layer. Since AAO is a porous material, it is considered a parallel combination of two capacitors, filled with alumina and air, respectively, as shown in Equation (3) [21]:

(3) $$C=\;\frac{\varepsilon_{alumina}A(1-\alpha )}{d}+\;\frac{\varepsilon_{air}A\alpha }{d}$$

where α is the porosity of the AAO nanofilm. Under external pressure, the elastic PDMS layer is deformed and conformally pressed into the platinum-coated AAO nanopores, thereby displacing the internal air and inducing a detectable alteration in the baseline capacitance. The dielectric constant of PDMS is typically between 2.6 and 3.0 [29,30], which is much higher than that of air (1.0). Therefore, the capacitance of the AAO pressure sensor increases accordingly. As the pressure continues to rise, it becomes increasingly difficult for the PDMS to enter the pores further, eventually leading to saturation at 73 kPa. The sensitivity of a capacitive pressure sensor is defined by Equation (4) below [31,32,33,34,35]:

(4) $$Sensitivity=\;\frac{C-C_{0}}{P-P_{0}}$$

where *C* and *P* represent the capacitance and pressure at a given measurement point, and *C*_0_ and *P*_0_ represent the capacitance and pressure at the minimum measured pressure. Based on Equation (4), the proposed Al-AAO/PDMS pressure sensor exhibits a good sensitivity of 5.56 pF/kPa up to 73 kPa.

**Figure 8:**
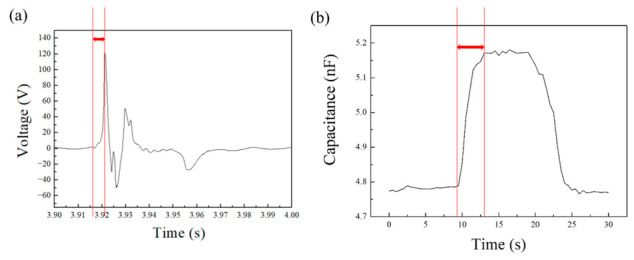
Response time curves of the hybrid Al-AAO/PDMS sensor under (**a**) dynamic contact-separation testing and (**b**) static pressure sensing. The red vertical lines and horizontal arrows indicate the response time required for induction.

Figure 8 shows the response time characteristics of the Al-AAO/PDMS hybrid pressure sensor to evaluate its operational agility across different sensing modalities. As demonstrated in Figure 8a, the triboelectric nanogenerator mode exhibits an exceptionally rapid response time of 50 ms when subjected to a dynamic pressure of 73 kPa delivered by the pneumatic cylinder. In contrast, Figure 8b shows that the static capacitive mode requires 3 s for the capacitance signal to stabilize at its peak value under an equivalent static mechanical load. This pronounced discrepancy between the two response times originates from the fundamentally distinct physical mechanisms that govern the dynamic and static transduction pathways. The rapid 50 ms response of the triboelectric mode is determined by the instantaneous nature of contact electrification and electrostatic induction, which occur immediately upon the initial physical impact and rapid macroscale compression of the bilayer interface. Conversely, the longer 3 s stabilization time observed in the capacitive mode is strictly restricted by the viscoelastic nature of the polydimethylsiloxane layer and the severe geometric confinement of the nanoporous framework. Under static loading, the elastic polymer must physically deform and conformally flow into the narrow cylindrical nano-channels to displace the internal air. This continuous infiltration process is governed by viscoelastic relaxation and localized fluidic resistance within the restricted nanopores, which naturally prolongs the time required for the polymer to reach its structural deformation limit and stabilize the total dielectric output. Despite the slower transition time of the capacitive signal, this dual-mode integration successfully delivers a highly complementary sensing framework. The swift triboelectric response efficiently captures transient mechanical impacts, while the stable capacitive output compensates for the inherent limitations of conventional nanogenerators by handling sustained static pressure holding.

Table 1 summarizes the performance of various voltage-output pressure sensors, including both piezoelectric and triboelectric operation modes [36,37,38,39,40]. The dynamic triboelectric voltage sensitivity of 1.05 V/kPa achieved by the proposed hybrid sensor significantly outperforms the values reported for the conventional single-mode piezoelectric and triboelectric pressure sensors in the previous literature. This prominent enhancement in electrical performance is primarily driven by the strategic integration of the nanoporous anodic aluminum oxide layer as the active triboelectric material. Unlike traditional flat thin films or chemically modified surfaces that suffer from restricted contact boundaries under mechanical load, the intrinsic self-organized topography of the anodic aluminum oxide provides a dense matrix of vertical nano-channels. Under external pressure, the highly elastic PDMS film undergoes localized deformation and conformally fills into these open nanopores, which drastically expands the effective contact area at the electrification interface and leads to an elevated surface charge density during contact-separation cycles. Beyond the microstructural advantages of the porous oxide template, the exceptional sensitivity is further supported by the systematic optimization of the elastomeric polymer thickness. Controlling the polydimethylsiloxane thickness at exactly 450 μm establishes a critical physical equilibrium between robust dielectric charge accumulation within the bulk polymer and efficient charge extraction across the conductive electrode interface. When the thickness is too small, the charge storage capacity of the dielectric layer remains insufficient, whereas an excessive thickness introduces an increased internal resistance that severely impedes the induction of external current. Therefore, this synergistic combination of a high-surface-area nanoporous architecture and an optimized polymer thickness allows our streamlined bilayer sensor to deliver an outstanding voltage response alongside a rapid response time of 50 ms, all while maintaining a remarkably short fabrication workflow of approximately 2 h.

On the other hand, Table 2 compiles the comparison of various capacitive-type pressure sensors [31,32,33,34,35]. The exceptional capacitive performance of Al-AAO/Pt can be fundamentally explained by the microscale thickness and nanoporous structure of the AAO layer. According to the parallel plate capacitor model, the total capacitance is inversely proportional to the dielectric separation distance, meaning that the ultra-thin oxide matrix with a thickness of only 5.9 μm inherently establishes a remarkably large baseline capacitance. Consequently, even a minor physical perturbation at the interface triggers an exceptionally pronounced absolute capacitance modification that drastically amplifies the overall sensing sensitivity. Furthermore, this outstanding sensitivity relies heavily on the profound dielectric modulation occurring within the localized nanoporous channels. Under external mechanical loading, the highly elastic polydimethylsiloxane layer progressively deforms and fills the open vertical pores of the oxide framework. This mechanical infiltration leads to the incremental displacement of the internal air, which possesses a low dielectric constant of 1.0. As the air is substituted by the entering polymer material which features a significantly higher dielectric constant between 2.6 and 3.0, the effective composite dielectric constant of the parallel aluminum oxide and polymer network rises dramatically. This penetration-driven material substitution induces a massive and highly resolvable alteration in capacitance over the entire testing range. In addition, owing to the rapid one-step HPA fabrication, the preparation time of the proposed hybrid device is limited to approximately 2 h, which is significantly shorter than that of reported platforms required in complex material synthesis or photolithography.

To evaluate the long-term operational durability and mechanical stability of the Al-AAO/PDMS dual-mode pressure sensor, a continuous cyclic loading test was conducted for 5000 cycles under a periodic vertical impact force of 15 N operating at a dynamic frequency of 7 Hz. As demonstrated in Figure S2, the triboelectric open-circuit voltage output exhibits no noticeable degradation throughout the extended testing period, maintaining a remarkably stable peak-to-peak amplitude after an initial charge stabilization phase where the contact surfaces reach electrostatic saturation. This outstanding durability is primarily attributed to the excellent complementary mechanical profiles of the selected material pairs. The polydimethylsiloxane layer serves as a highly elastic counterpart that undergoes fully reversible deformation without experiencing structural fatigue or permanent plastic deformation under repeated mechanical impact. Concurrently, the underlying AAO film features exceptionally high mechanical hardness and structural rigidity, which effectively prevents material wear or surface abrasion at the microscale contact interface. Consequently, the structural integrity of the nanoporous matrix and the conformal platinum coating remains perfectly preserved, confirming the outstanding operational reliability and robustness of the streamlined bilayer architecture for sustainable real-world applications.

The proposed Al-AAO/PDMS dual-mode pressure sensor holds great promise for future applications, driven by its dual-mode sensing capability, streamlined architecture, and efficient one-step anodization process. A range of sensing from 1 to 73 kPa is highly suitable for a wide range of practical applications, spanning from physiological monitoring to robotic tactile sensing [24]. The low-pressure regime from 1 to 20 kPa is well-suited for subtle tactile sensations and physiological signal monitoring, such as pulse wave detection [24] and gentle human tactile discrimination [25]. The medium-pressure regime from 20 to 73 kPa effectively covers daily human activities and industrial tasks, including object grasping by robotic hands [26], foot pressure distribution monitoring [27], and impact force detection during locomotion [28]. In summary, the comprehensive evaluation of the Al-AAO/PDMS framework demonstrates outstanding performance parameters for both high-sensitivity dynamic impact recording and reliable static pressure retention tracking across diverse practical scenarios. Nevertheless, it is essential to note that the current operational configuration is restricted to dual-mode independent characterization, where the dynamic triboelectric pulses and static capacitive modulations are recorded sequentially or on separate testing platforms to eliminate cross-talk artifacts. To overcome this limitation and achieve true simultaneous real-time detection on a single shared electrical loop, future work will focus on integrating advanced peripheral signal processing circuitry. Implementing active frequency-selective filters, such as high-pass and low-pass networks, alongside sophisticated mathematical decoupling algorithms will enable the clean separation of high-frequency transient triboelectric voltage signals from slow static capacitive variations. This hardware and software coordination will successfully allow for a single unified readout channel, thereby unlocking the full potential of this streamlined bilayer architecture for next-generation smart tactile electronics and autonomous wearable systems.

## 4. Conclusions

In this work, a streamlined Al-AAO/PDMS dual-mode pressure sensor integrating dynamic triboelectric and static capacitive sensing is proposed. Directly growing the nanoporous AAO layer by a rapid one-step anodization at room temperature reduces structural complexity and total fabrication time. As a core innovation, the AAO plays a dual role by acting as an active triboelectric layer that expands the contact area to increase power output, while concurrently serving as a capacitive dielectric layer that enables PDMS penetration to detect static loads. Under an optimized polymer thickness of 450 μm, the device delivers an open-circuit voltage of 118.4 V and a short-circuit current of 66.2 μA. Across a wide sensing range of 1 to 73 kPa, the sensor demonstrates a high dynamic sensitivity of 1.05 V/kPa with a 50 ms response time alongside a static capacitive sensitivity of 5.56 pF/kPa. This robust dual-mode capability successfully overcomes the transient limitations of traditional TENGs, showing great potential for robotic grasping and physiological monitoring.

## Figures and Tables

**Table 1:** Comparison of voltage-output pressure sensors.

Ref.	Materials	Mechanism	Process/Time	Sensitivity	Response Time
[36]	SiC/Si	Piezoelectric	CVD and photolithography/NA	1.71 × 10^−5^ VkPa^−1^ (1.71 mV/bar)	NA
[37]	PDMS and PVDF/MXene	Piezoelectric	Electrospinning and casting/About 3 days	0.059 VkPa^−1^	NA
[38]	Styrene-ethylene-butylene-styrene and PDMS	Triboelectric	Chemical synthesis/About 13 h	0.076 VkPa^−1^ (0.76 V/N)	NA
[39]	Parylene-Cu-PAN	Triboelectric	Chemical synthesis/About 4.5 h	0.344 VkPa^−1^	NA
[40]	PTFE and tungsten needles	Triboelectric	Printed circuit board process and hot melt adhesive/NA	0.00835 (kPa^−1^)	54 ms
This work	Al-AAO/PDMS	Hybrid triboelectric and capacitive	Anodization and casting/About 2 h	1.05 VkPa^−1^	50 ms

NA: Not Available.

**Table 2:** Comparison of capacitive pressure sensors.

Ref.	Materials	Mechanism	Process/Time	Sensitivity
[31]	Si, SiO_2_, Al and glass	Capacitive	Photolithography and etching/NA	0.072 pFkPa^−1^
[32]	Si, SiO_2_ and Al	Capacitive	Photolithography, etching and PECVD/NA	0.057 pFkPa^−1^
[33]	PDMS-AgNPs	Capacitive	Molding and hardening/NA	0.035 pFkPa^−1^
[34]	AAO	Capacitive	Anodization/NA	0.3 pFkPa^−1^
[35]	CNTs, MXene and PDMS	Capacitive	Electrospinning and chemical synthesis/About 80 h	0.091 kPa^−1^
This work	Al-AAO/PDMS	Hybrid triboelectric and capacitive	Anodization and casting/About 2 h	5.56 pFkPa^−1^

NA: Not Available.

## Data Availability

Data are presented in the coauthors’ research results, and the schematic drawing is available upon request.

## Supplementary Materials

The following supporting information can be downloaded at: https://www.mdpi.com/article/10.3390/nano16120771/s1, Figure S1: The actual photograph of the fully assembled Al-AAO/PDMS device; Figure S2: Open-circuit voltage output of the Al-AAO/PDMS dual-mode pressure sensor during the 5000-cycle durability test.
