# Supplementary material for: Dual-Mode Triboelectric and Capacitive Pressure Sensor Based on Anodic Aluminum Oxide
Source: Nanomaterials (Basel). 2026 Jun 19;16(12):771. doi: 10.3390/nano16120771 (PMC13304531; doi:10.3390/nano16120771)
Supplement: Supplementary file 1 [file nanomaterials-16-00771-s001.zip › nanomaterials-4312546-supplementary.pdf]

nanomaterials

Supplementary Materials

Dual-Mode Triboelectric and Capacitive Pressure Sensor Enabled by Anodic Aluminum Oxide

Chung-Yu Yu, Chia-Wei Hung, Chin-An Ku, Geng-Fu Li, Cheng-Hao Chiu and Chen-Kuei Chung*

Department of Mechanical Engineering, National Cheng Kung University, Tainan 701, Taiwan;
* Correspondence: ckchung@mail.ncku.edu.tw

After demolding, the PDMS film was placed onto the Pt-sputtered AAO substrate, completing the sensor fabrication. The actual optical photograph of the fully assembled dual-mode Al-AAO/PDMS pressure sensor was shown in Figure S1. The aluminum substrate and the top platinum layer on the AAO serve as two parallel metal electrodes for the capacitor, with the copper tape serving solely as a conductive wire to facilitate subsequent electrical measurements. Meanwhile, the AAO and PDMS act as the two triboelectric layers for the TENG. In addition, the AAO also functions as a porous dielectric layer for the capacitor, allowing the PDMS to penetrate the pores and modulate the capacitance.

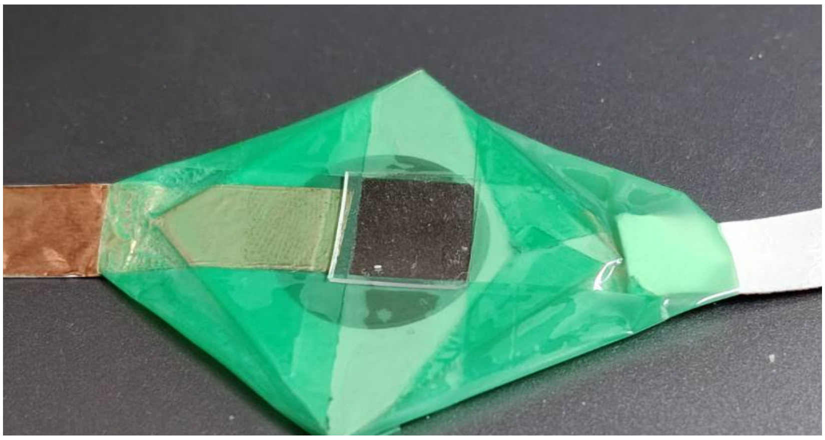

Figure S1 The actual photograph of the fully assembled Al-AAO/PDMS device.

To evaluate the long-term operational durability and mechanical stability of the Al-AAO/PDMS dual-mode pressure sensor, a continuous cyclic loading test was con-ducted for 5000 cycles under a periodic vertical impact force of 15 N operating at a dynamic frequency of 7 Hz. As demonstrated in Figure S2, the triboelectric open-circuit volt-age output exhibits no noticeable degradation throughout the extended testing period, maintaining a remarkably stable peak-to-peak amplitude after an initial charge stabilization phase where the contact surfaces reach electrostatic saturation. This outstanding durability is primarily attributed to the excellent complementary mechanical profiles of the selected material pairs. The polydimethylsiloxane layer serves as a highly elastic counterpart that undergoes fully reversible deformation without experiencing structural fatigue or permanent plastic deformation under repeated mechanical impact. Concurrently, the underlying AAO film features exceptionally high mechanical hardness and structural rigidity, which effectively prevents material wear or surface abrasion at the microscale contact interface. Consequently, the structural integrity of the nanoporous

 https://doi.org/10.3390/nano16120771

matrix and the conformal platinum coating remains perfectly preserved, confirming the outstanding operational reliability and robustness of the streamlined bilayer architecture for sustainable real-world applications.

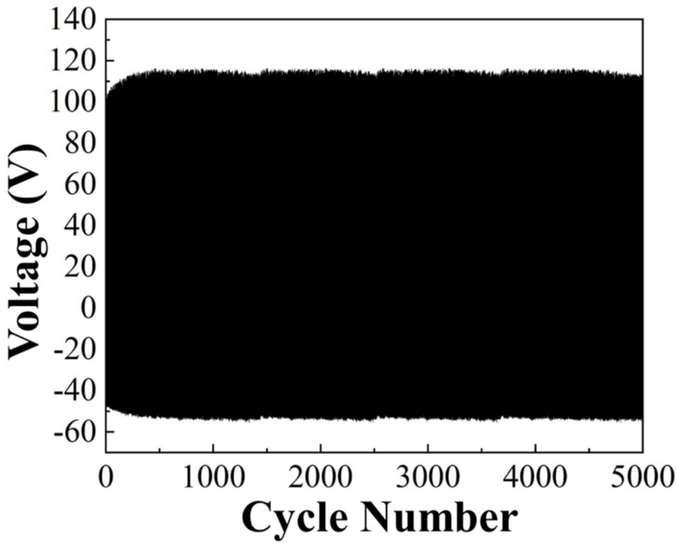

Figure S2. Open-circuit voltage output of the Al-AAO/PDMS dual-mode pressure sensor during the 5000-cycle durability test.
